# Detection of Alzheimer’s Disease Based on Cloud-Based Deep Learning Paradigm

**DOI:** 10.3390/diagnostics13162687

**Published:** 2023-08-15

**Authors:** Dayananda Pruthviraja, Sowmyarani C. Nagaraju, Niranjanamurthy Mudligiriyappa, Mahesh S. Raisinghani, Surbhi Bhatia Khan, Nora A. Alkhaldi, Areej A. Malibari

**Affiliations:** 1 Department of Information Technology, Manipal Institute of Technology Bengaluru, Manipal Academy of Higher Education, Manipal 576104, India; 2 Department of Computer Science and Engineering, R V College of Engineering, Bengaluru 560059, India; 3 Department of Artificial Intelligence and Machine Learning, BMS Institute of Technology and Management, Bengaluru 560064, India; 4 College of Business, Texas Woman’s University, Denton, TX 76204, USA; mraisinghani@twu.edu; 5 Department of Data Science, School of Science, Engineering and Environment, University of Salford, Manchester M54WT, UK; 6 Department of Computer Science, College of Computer Science and Information Technology, King Faisal University, Al Ahsa 31982, Saudi Arabia; 7 Department of Industrial and Systems Engineering, College of Engineering, Princess Nourah Bint Abdulrahman University, P.O. Box 84428, Riyadh 11671, Saudi Arabia

**Keywords:** Alzheimer’s disease, convolution neural network, deep learning, GoogLeNet

## Abstract

Deep learning is playing a major role in identifying complicated structure, and it outperforms in term of training and classification tasks in comparison to traditional algorithms. In this work, a local cloud-based solution is developed for classification of Alzheimer’s disease (AD) as MRI scans as input modality. The multi-classification is used for AD variety and is classified into four stages. In order to leverage the capabilities of the pre-trained GoogLeNet model, transfer learning is employed. The GoogLeNet model, which is pre-trained for image classification tasks, is fine-tuned for the specific purpose of multi-class AD classification. Through this process, a better accuracy of 98% is achieved. As a result, a local cloud web application for Alzheimer’s prediction is developed using the proposed architectures of GoogLeNet. This application enables doctors to remotely check for the presence of AD in patients.

## 1. Introduction

The image modality structural magnetic resonance imaging (MRI) for deep learning techniques has gained lot of importance within the research community. Many recent studies state that important feature extraction can be performed automatically using convolutional neural networks (CNNs), and it has ability to handle large image data. Neuroimaging techniques, such as magnetic resonance imaging (MRI) and positron emission tomography (PET), have undergone significant advancements in recent years. These techniques play a crucial role in the identification of structural and molecular biomarkers associated with Alzheimer’s disease (AD). MRI allows for detailed imaging of brain structures, while PET enables the visualization and quantification of specific molecular targets relevant to AD pathology [1]. These neuroimaging techniques contribute to our understanding of AD and aid in its diagnosis and management. The rapid development of neuroimaging techniques has presented challenges in effectively integrating large-scale, multidimensional neuroimaging data. As a result, there has been a surge of interest in employing computer-aided machine learning methods for comprehensive analysis. Promising pattern analysis techniques, such as linear discriminant analysis (LDA), linear program boosting method (LPBM), logistic regression (LR), support vector machine (SVM), and support vector machine-recursive feature elimination (SVM-RFE), have been employed in the field. These methods demonstrate potential in the early detection of AD and predicting the progression of the disease.

CT scans offer quick scanning times and provide clear images, making them suitable for examining various diseases. However, the resolution of the middle lobe is relatively low, which can lead to misdiagnosis of mild cognitive impairment (MCI) as a typical age-related phenomenon. PET imaging, on the other hand, enhances both sensitivity and resolution. To reduce the influence of tissue attenuation on the image, transmission scanning technology is employed in the imaging process. This technology aims to minimize the impact and distortion caused by the attenuation of tissues. On the other hand, magnetic resonance imaging (MRI) is widely utilized to visualize and examine the internal structures of the human body. It provides detailed and high-resolution images of various body tissues and organs. The utilization of rapidly changing gradient magnetic fields significantly enhances the speed of MRI scans. This advancement enables quicker image acquisition and reduces the overall scanning time. Moreover, MRI exhibits excellent resolution when imaging soft tissues, allowing for detailed visualization of anatomical structures and abnormalities. Importantly, MRI does not involve the use of ionizing radiation, making it a safe imaging modality with no associated radiation-related harm to the human body. In mental health trials, the most beneficial approach is computational neuroscientific, and it is very helpful in health decisions [1]. The number of people with dementia is estimated to be 151 million by the year 2050 [2]. The human states that are not normal can be monitored through this field of study. One of the main causes of dementia is Alzheimer’s disease (AD). AD is a degenerative brain condition characterized by a gradual decline in cognitive abilities. As a result, significant efforts have been dedicated to devising approaches for early detection, particularly during the pre-symptomatic stages, with the aim of impeding or halting the progression of the disease.

Clinical Dementia Rating [3] and Global Deterioration Scale [4] are the clinical tools used in assessment for tracing of disease. In order to assess, 24 h data need to be collected from sensor-based systems attached to patients. The field of AD diagnosis has witnessed increased interest in computer vision with the rapid advancements in artificial intelligence. To address the challenges mentioned previously, researchers are increasingly turning to deep learning, a burgeoning field within machine learning. Deep learning techniques enable the direct utilization of raw neuroimaging data, allowing for the generation of features through dynamic learning processes. This approach, often referred to as “on-the-fly” learning, has gained significant traction in large-scale, high-dimensional medical imaging analysis. Notably, deep learning methods, including convolutional neural networks (CNN), have demonstrated superior performance compared to traditional machine learning approaches. This has further contributed to the growing interest in deep learning for advancing medical imaging analysis. Deep learning, a prominent branch of machine learning, has emerged as the most popular approach to address the limitations of traditional methods. Particularly, deep learning technology has gained significant traction in medical imaging, allowing for the automatic extraction of features from medical images to facilitate AD detection. This innovative application has shown promising results in recent years. For imaging, the deep learning methods are frequently used for data analysis from neuroimaging [5] and to detect Alzheimer’s disease. In recent work, biomarkers for genetics are used in deep learning to diagnose AD. Positron emission tomography and magnetic resonance imaging are also used to articulate molecular biomarkers and structure in advanced neuroimaging techniques. A few machine learning algorithms like linear program boosting method and support vector machine recursive feature elimination have given good results for early prediction of AD [6], and this has caused quick progress in neuroimaging. In general, four steps are followed in machine learning classification: a. extraction of features, b. selection, c. reduction in dimensionality, d. classification based on feature selection. Our proposed work concentrates on the analysis of Google Net architecture of Convolutional Neural Networks architecture for training and classification for predicting the Alzheimer’s disease using local cloud-based solutions.

Brain imaging techniques play a crucial role in non-invasively assessing the function, structure, and pharmacology of the brain for Alzheimer’s disease (AD). These techniques provide valuable insights into the brain’s activities and help in predicting the progression of AD [7]. The imaging techniques are usually separated into two groups, namely functional imaging and structural imaging [8]. Functional imaging discusses the activities performed by the brain, and structural imaging discusses the brain’s structure, including neurons, glial cells, synapses, etc. [9]. The commonly employed neuroimaging techniques for Alzheimer’s disease (AD) include magnetic resonance imaging (MRI), positron emission tomography (PET), and MRI biomarkers specifically associated with AD. These techniques enable the visualization and assessment of brain structure, function, and specific biomarkers relevant to AD. They provide valuable diagnostic information and contribute to our understanding of the disease.

The magnetic resonance imaging (MRI) technique utilizes magnetic fields and radio waves to generate high-quality and high-resolution images of brain structures in both 2D and 3D formats. Unlike other imaging methods such as X-rays, MRI does not involve the use of ionizing radiation. Instead, it employs harmless magnetic fields and radio waves to create detailed images of the brain’s anatomy. This makes MRI a safe and non-invasive technique for imaging the brain. The structural MRI is the commonly used MRI, imaging brain volumes in vivo to notice brain degeneration. Brain degeneration can be defined as expected progressive module of AD [10,11]. Functional magnetic resonance imaging (fMRI) is the extensively practiced method, which gauges the human cortex and identifies brain topography. fMRI offers helpful data about the activities of the human brain. Single-photon emission computed tomography (SPECT) is delicate for the early examination of modifications in cerebral blood flow. It is more economical compared to other techniques [12]. Based on many studies during AD examination, SPECT specifically computes the cerebral perfusion. A recent study scrutinized 116 patients. A total of 67 individuals had noticeable other neurological concern, 26 of those had evident non-Alzheimer’s dementia, and 23 of those were labeled as age-matched controls [13]. Cerebral perfusion, cerebrospinal fluid (CSF)-tau, and cognitive proteins examination was carried out. The themes were categorized into control and dementia case. Cognitive functions and functional conditions were classified based on the Cambridge Cognitive Examination, Mini-Mental State. The CSF-tau protein SPECT is additionally suitable for the inspection of AD. The positron emission tomography (PET) imaging method makes use of radiotracers, and the brain’s activities are examined as radioactive spheres.

By utilizing radioligands C-PMP and C-MP4A, acetylcholinesterase was experimental. These results specify a lessening in the temporal lobes of the AD [14]. Identical refuse was experiential along with MCI, which ultimately evolved to AD. A temporoparietal hypoperfusion intuition was experimental in mainly the AD. False-positive results, for clinical purposes, render SPECT problematic; by disparity, use of FP-CIT SPECT and neuroreceptors are additionally helpful and convenient. This allows visualization of incongruity in the nigrostriatal dopaminergic neurons and estimation of the direction, position, and anisotropy in the brain. Though significant studies have been conducted to recognize CSF-tau biomarker and amyloid levels, there exists a deficiency in unanimous conclusions that hinder diffusion tensor imaging (DTI) from being incorporated as an unfailing method for understanding CSF biomarkers [15]. AD MRI biomarkers are considered as one of the medical symbols with the aim of deliberating precisely [16]. Biomarkers as objects are defined by the Chemical Safety International Program as an architecture that is measured and where the existence of a disarray can be completed [17]. The properties with respect to AD biomarkers are as follows: firstly, they are able to recognize essential characteristics of AD’s neuropathology; secondly, they are able to attest neuropathologically confirmed AD cases; thirdly, they are efficient, able to recognize initial AD and to differentiate AD with a dissimilar outline of dementias; fourth, they are non-invasive, reliable, inexpensive, and easy to implement. Biomarkers that also help depict AD: neuroimaging biomarkers, biochemical, and genetic [18]. MRI biomarkers are measured because of their huge impending in AD detection. Two actions can conclude structural connectivity with functional connectivity where they consist of additional authority and resources of AD; however, they silently engage in authorization and regulation to guarantee clinical utility. This indicates that when hippocampus volume is considered, the structural MRI is the most efficient and nearly always utilized MRI biomarker.

Considering 11 [19,20,21,22,23,24,25,26,27,28,29] papers, Table 1 gives the top results of guess of MCI to conversion (AD) and/or diagnostic classification. Only binary classification outcomes are compared. Accuracy is a quantity utilized constantly in 11 publications. Conversely, one metric performance uniqueness of the algorithm. Sample sizes, composition, and scan numbers examined are noted jointly since accuracy is responsive to unbalanced distributions.

Deep learning approaches involve enormous data, which attain preferred levels of concert accuracy. The original image values were decoded using an autoencoder (AE), which was similar to the original image, including input, consuming the imperfect neuroimaging data. Current progress in technology of usability and availability accompanies the conventional hospital-centered concept with the likelihood of collecting info from the user’s day-to-day personalized interventions. Sensor devices have the benefit of not involving any relations effort of the patient. Thus, its purpose suggests an ample series of possibilities, with fundamental daily living actions or as the detection of falls [30] or leaving-bed episodes during nighttime [31]. Wearable sensors discovered for numerous dissimilar reasons. Gietzelt [32] composed data by a tri-axial accelerometer permissible to distinguish involving patients who consequently undergo falls, increasing the likelihood of building evaluation of being a fall risk. By considering 319 non-demented elderly people, sample analogous results were established through Van Schooten [33]. Supervising of behavioral disturbances is one more exciting possible applications of wearable accelerometers.

Quite a lot of works revealed that actigraphy is capable of recognizing apathy that represents the majority of recurrent neuropsychiatric symptoms [34,35,36]. Moreover, Goerss et al. [37] calculated patients in the firm phase with sensors for seven days in two nursing homes. Composed facts were utilized that estimate an AMS, which was certainly associated with the total intensity. Furthermore, AMS was considerably connected with precise abnormal behaviors and apathy mannerisms. The analyzed consequences advise that accelerometry is supportive to build individual predictions and to estimate the reaction potential treatments. Furthermore, current studies illustrate the usefulness of accelerometry, which differentiates dementia subtypes—Parkinson’s disease dementia and dementia with Lewy body—recognizing important diversities in seven conventional gait features [38]. Modification to gait parameters has been discovered as a risk factor for dementia. Gillain et al. and Kirste et al. [39] calculated each day deeds in 23 patients and also their unimpaired partners from side to side, and the data were acquired by ankle-mounted tri-axial accelerometers. Mutually, clusters were distinguished with taxonomy accurateness of 91%. Fascinatingly, attainment in the Mini-Mental Examination [40], where a concise test is regularly used for review clinical progression, was considerably associated with motion features, which proposes a possible utility to recognize the phase of the disease. Furthermore, current studies show promising effectiveness of accelerometry. Circadian rhythm turbulence is a regular ruling in AD dementia and is estimated with accelerometry [41]. In a recent work, 189 unimpaired member cerebrospinal fluid analysis examined circadian rhythms using actigraphic data, which tolerate the survival of preclinical AD to be clear [42]. Independent of age or sex, improved intradaily variability was allied with preclinical AD. In addition, Li et al. [43] illustrated 1097 cognitively unimpaired seniors who experienced a broad cognitive study, as well as the estimation of daily motor movement. The frequency of dementia was extremely connected through degraded motor FR, a good number of devices that normalize patterns of day-to-day motor activity fluctuations (95% CI 1.15–1.49, HR 1.31, *p* < 0.0001). Moreover, extra degraded regulation was connected with quicker cognitive refuse, signifying that it predicts quick clinical progression. Fascinatingly, these conclusions headed the diagnosis involves that perturbations of FR perceived by actigraphy earlier than clinical onset. At the outline, AD appears to go together with the modification with motor movement features and a sufficient approach to evaluate.

A number of earlier studies revealed how to obtain the benefit of accelerometers that achieve an expenditure investigation [44] by utilizing those integrated smartphones [45]. Until now, accelerometry was inspected to sense the occurrence of AD, either in the preclinical state or in clinically affected subjects, but not often to identify diverse clinical stages, where there is capital significance to identify clinical progression. Using sensor-based devices, various preceding works illustrate the organization of AD patients with three functional levels [46,47], but CNN was not useful with the dementia staging idea until now. El Maachi et al. [48] proposed a CNN-based method that identifies Parkinson’s disease examining data of gait. Bringas et al. [49], civilizing the outcome of Nieto-Reyes et al. [46], gave an initial advance to the problem using CNN. Adding to introduce preprocessing structure for data, this paper utilizes a permanent architecture and its parameters supporting exclusively on accuracy, attaining enhanced and extra objective results comparing earlier methods applied.

### 1.1. Motivation

Given the current circumstances, there is often a disparity between human intuition and standard measurements. To address this issue, we must embrace unconventional and computationally intensive approaches, such as deep learning. Deep learning techniques are increasingly utilized in disease prediction and visualization, providing insightful and personalized treatment recommendations. This advancement not only enhances patients’ quality of life, but also assists physicians in making informed treatment decisions and enables health economists to conduct thorough analyses. Relying solely on medical reports can cause radiologists to overlook other disease conditions, as they typically consider only a limited number of causes and conditions. The objective at hand is to identify the gaps in knowledge and potential opportunities associated with deep learning architecture.

### 1.2. Contribution

In our research, we focus on identifying individuals affected by Alzheimer’s disease and aim to detect potential cases at an early stage. The datasets for Alzheimer’s disease are obtained from ADNI and serve as the training data for our deep learning architecture, specifically GoogLeNet. By leveraging this powerful architecture, we are able to effectively differentiate affected individuals with a high level of efficiency and speed.

The flow of this paper is organized as follows. reviews previous works on the prediction and analysis of Alzheimer’s disease. Section 2 describes our GoogLeNet architecture of CNN for prediction of Alzheimer’s. Section 3 shows the experimental results between proposed and existing methods. Section 4 discusses the results presents the conclusions.

## 2. Materials and Methods

Detection of Alzheimer’s disease at the early stage shows a vital role in averting and supervising its progress. A proposed outline for the early detection of Alzheimer’s disease is presented. In this section, proposed framework details, the dataset used, deployment of the proposed solution in the local cloud, and the algorithmic flow of the proposed system are explained.

### Dataset

The data used for this paper were collected from the Alzheimer’s Neuroimaging Initiative (ADNI) Dataset, which can be accessed at http://adni.loni.usc.edu (accessed on 20 March 2023). The ADNI repository was established in 2003 through a public–private partnership. The primary objective of ADNI was to investigate the combination of sequential MRI, CT, and PET scans and neurophysical evaluations to study the progression of mild cognitive impairment and early-stage Alzheimer’s disease. Training data were also collected in ADNI for 2D. Images in the neuroimaging initiative link will be in DICOM format. It contains image portrayals like Axia, Sagittal, and Coronal. A total of 300 patient datasets were collected in ADNI, and they are divided into 4 classes, i.e., LMCI, AD, NC, and EMCI. LMCI comprises 3460 images, EMCI comprises 5817 images, NC comprises 6775, and finally images from the AD class comprise 5764 images.

The size of the images is 256 × 256, and Table 2 portrays demographic data from ADNI. The details steps involved in the proposed solution are given in the algorithm below, taking into consideration image format conversion, the training model using GoogLeNet, and cloud deployment including the classifier.

**Table 2:** Three-hundred subjects’ demographic data.

Alzheimer Stages	AD	LMCI	NC	EMCI
Subject number	75	75	75	75
Male/female	21/54	43/32	32/43	51/24
Age (mean ± STD)	7 5.95 ± 0.91	77.44 ± 1.33801	75.68 ± 0.469617	76.08 ± 0.89684

**Algorithm 1**: Proposed ModelStep 1: Input the Dicom image from MRI scansStep 2: Pre- Process the images and converting them to jpeg format and removing noiceStep 3: Reformat the images and resize them from 256 × 256 to 224 × 224Step 4: Images are classified into EMCI, NC, LMCI and AD.Step 5: GoogleNet Model method uses transfer learning technique for training 268 pre trained images and classify input images as AD and Normal caseStep 6: A web based application is designed to assist docters to check AD from remote place using local application and Microsoft Azure plaform

The proposed work comprises four stages as show in Algorithm 1:

Stage 1—Data Procurement and Preprocessing Stage: Data images were collected from Alzheimer’s disease neuroimaging initiative through the link: http://adni.loni.usc.edu/ (accessed on 20 March 2023), and training data were also collected in ADNI for 2D. Images in the neuroimaging initiative link will be in DICOM format. It contains image portrayals like Axia, Sagittal, and Coronal. A total of 300 patient datasets were collected in ADNI, and they are divided into 4 classes i.e., LMCI, AD, NC and EMCI. LMCI comprises 3460 images, EMCI comprises 5817 images, NC comprises 6775, and finally images in the AD class comprise 5764 images. The size of the images is 256 × 256, and Table 2 portrays demographic data from ADNI.

**Figure 1:**
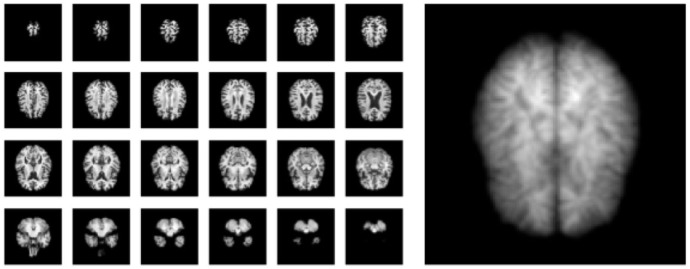
Brain MRI sample.

**Figure 2:**
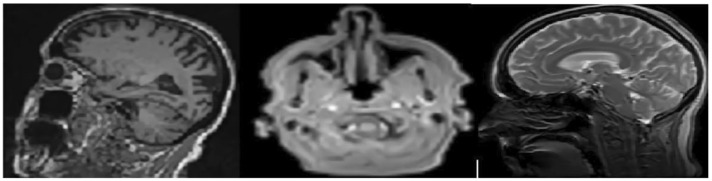
View of AD patient from left to right.

Figure 1 shows 2D imaging slice of MRI and also formation of 3D using 2D image slices, and Figure 2 demonstrates slices hauled out from an MRI scan. For the proposed GoogLeNet CNN architecture, the input size is 224 × 224. The image size should be resized from 256 × 256 to 224 × 224.

This section may be divided by subheadings. It should provide a concise and precise description of the experimental results, their interpretation, as well as the experimental conclusions that can be drawn.

We resampled the data by deleting a few examples that are over-denoted classes, and replication was performed for under-denoted classes. All AD classes after resampling 6000 MRI images were retained. The data were then preprocessed by resizing and converting to a suitable format.

Stage 2—Image classification: In this stage, image are classified into EMCI, NC, LMCI, and AD. The proposed method uses the transfer learning technique, i.e., with the GoogLeNet model from pre-trained images. Using the ADNI pre-trained images, the proposed model is generated. In turn, the created trained model will be used for image classification.

**Figure 3:**
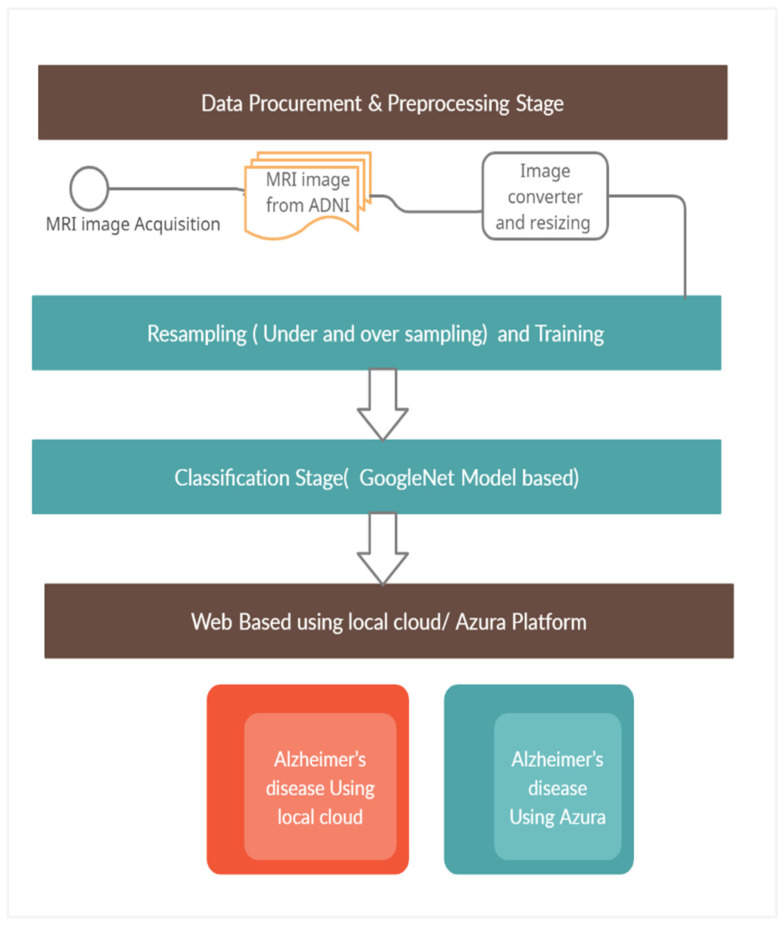
Proposed architecture.

Stage 3—A web-based application was designed to assist doctors to check AD from a remote place using a local based application and the Microsoft Azure platform. The developed solution is deployed in Microsoft Azure cloud as System as Service for accessing the web application remotely. The complete proposed solution is shown in Figure 3. A total of 27 layers are constituted in GoogLeNet architecture, including the pooling layer, and a total of nine modules are part of the inception. Sweeping window is exploited across the conv and pooling layer. Depth indicates the number of layers in the architecture. One of the procedures in deep learning is Transfer Learning, which is trained in this neural network.

## 3. Results and Discussion

In our research, we employ the principle of transfer learning for classifying medical images. Transfer learning involves training a neural network model on a similar problem before addressing the specific issue at hand. This approach offers several advantages:

(i) It leverages the pre-trained weights obtained from training millions of images in a database.
(ii) It reduces the time required for training a learning model.
(iii) It helps minimize errors in generalization.

**Figure 4:**
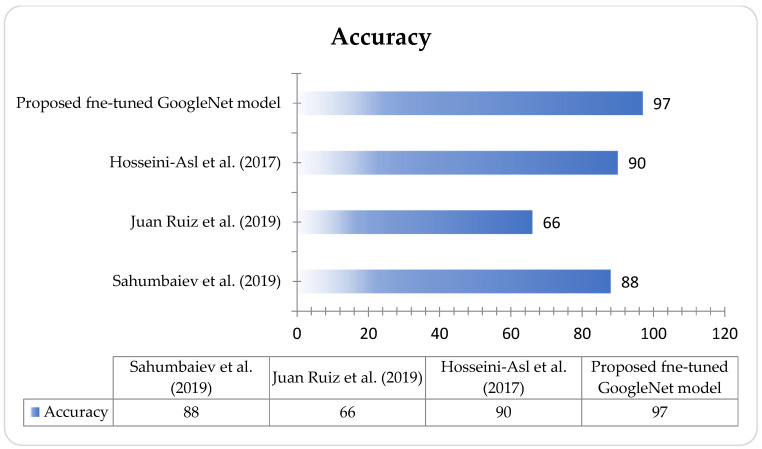
Accuracy comparison of proposed and existing models (Reference Sahumbaiev et al. (2019) [10], Juan Ruiz et al. (2019) [12], Hosseini-Asl et al. (2017) [9].

**Figure 5:**
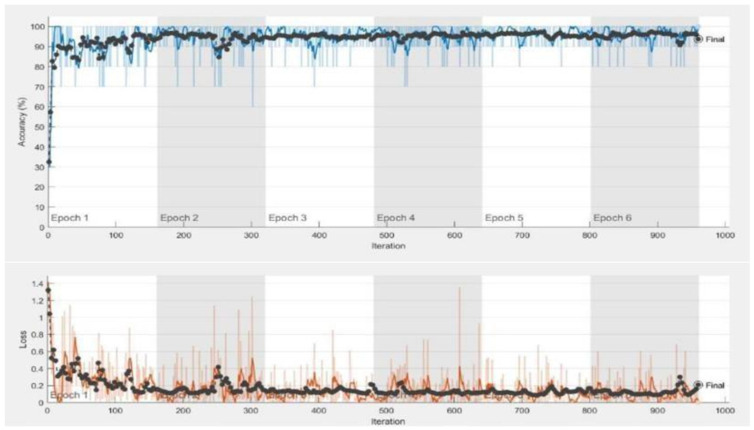
Training and validation accuracy of proposed model.

Consequently, we utilize the pre-trained GoogLeNet model for multi-class classification of MRI images. GoogLeNet is a convolutional neural network with an architecture consisting of 27 layers. To make it suitable for our medical image classification task, we perform a basic fine-tuning on the final layer of GoogLeNet. The fine-tuned GoogLeNet has 20,024,184 trainable parameters and zero non-trainable parameters. The specific tuning adjustments made to GoogLeNet are detailed in Table 3. The advantages of Transfer Learning are that its training time is much less and it reduces errors in generalization. Consequently, we utilized the pre-trained GoogLeNet model for multi-class classification. The tuning applied to the GoogLeNet model is depicted in Table 3. The multi-class image classification comparison with the proposed GoogLeNet model is shown in Figure 4. Figure 5 shows the training and validation accuracy of the proposed model using MatLab.

**Figure 6:**
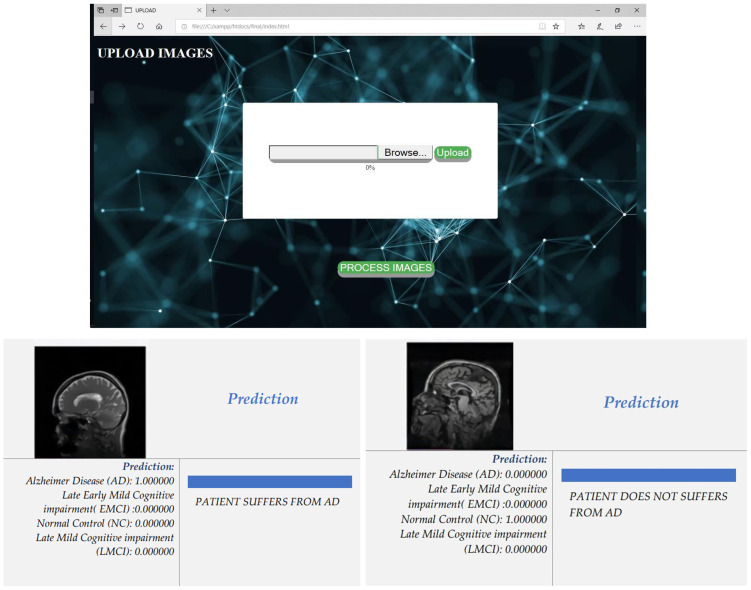
Upload Page to input image base and predication—AD/NC.

The local server is created in order to provide Software as a Service (SaaS), and the intention of designing the front end is to provide easy access to the system developed. Thematlab is used to develop, train, and validate the GoogLeNet model for the ADNI image base. The image will be inputted using the front end from MRI/CT, and the processing will be performed at the back end. In the back end, the image will be pro processed, and classification will be performed. In order to achieve this, we used html, css, js, php, and xampp. The snapshot of the local cloud-based solution is shown in Figure 6.

Table 4 and Figure 5 demonstrate the results of our study on multi-class medical image classification of AD stages. The fine-tuned GoogLeNet model we proposed achieved the highest accuracy of 98%. In comparison, Sahumbaiev et al. [10] achieved an accuracy of 88%, while Juan Ruiz et al. [41] had the lowest accuracy of 66.7%. Our proposed solution proves to be more efficient in this context. Additionally, we achieved promising accuracy for both binary and multi-class classification tasks.

## 4. Conclusions

This work introduces a framework that utilizes deep learning convolutional neural network (CNN) architectures, specifically employing transfer learning. The proposed method leverages the concept of transfer learning to benefit from pre-trained models. The GoogLeNet model is fine-tuned and employed for classification tasks, achieving an impressive accuracy of 98% in multi-stage Alzheimer’s disease (AD) classification. Additionally, the work proposes an Alzheimer’s checking web application based on the final architectures developed. The extension of the proposed solution is performed by developing a cloud-based solution for assisting doctors, and also the solution can be accessed remotely using the developed interface without any difficulty.

## Figures and Tables

**Table 1:** Top results of guess of MCI to conversion (AD) and diagnostic classification.

References	Modality	Data Processing/Training	Classifier	AD: NC acc.	SEN	SPE	cMCI:nMCI aa.	SEN	SPE	AD	cMCI	ncMCI	NC	Total
Li et al. (2014) [19]	MRI, PET	3D CNN	Logistic regression	92.97			76.21			198	167	236	229	830
Suk et al. (2014) [20]	MRI, PET	DBM	SVM	97.35	94.6	95.2	75.92 86.75 (MCI:NC)	48.04 95.37	95.23 65.87	93	76	128	101	398
Li et al. (2015) [21]	MRI, PET, CSF	RBM + drop out	SVM	91.4			57.4 76.21 (MCI:NC)			51	43	56	52	202
Suk et al. (2015) [22]	MRI, PET, CSF	SAE + sparse learning	SVM	98.8			83.3 90.7 (MCI:NC)			51	43	56	52	202
Liu et al. (2015) [23]	MRI, PET	SAE with zero masking	Softmax	91.4	92.32	90.42	92.1							
Suk and Shen (2013) [24]	MRI, PET, CSF	SAE	SVM	95.9			75.8			51	43	56	52	202
Cheng and Liu (2017) [25]	MRI, PET	3D CNN+ 2D CNN	Softmax	89.64	87.10	92.00				199			229	428
Vu et al. (2017) [26]	MRI, PET	SAE+ 3d CNN	Softmax	91.14						145			172	317
Liu et al. (2014) [27]	MRI, PET	SAE+ NN	Softmax	87.76	88.57	87.22	76.92	74.29	78.13	65	67	102	77	311
Choi and Jin (2018) [28]	MRI, PET	3D CNN	Softmax	96	93.5	97.8	84.2	81.0	87.0	139	79	92	182	494
Lu et al. (2018) [29]	PET	DNN + NN	Softmax	84.6	80.2	91.8	82.93	79.69	83.84	238	217	409	360	1224

**Table 3:** Tuning performed on GoogLeNet Model.

Model: “Sequential”		
Layer (Type)	Output Shape	Param
GoogleNet (functional)	(1,64,24,24)	20,024,184
Flatten (Flatten)	(1,4608)	0
dense (Dense)	(1,192,12,12)	4,710,612
dense_1 (Dense)	(1,480,6,6)	524,800
dense_2 (Dense)	(1,832,3,3)	131,528
dense_3 (Dense)	(1,1024,1,1)	32,996
dense_4 (Dense)	(1,10)	8
Trainable params: 25,424,128		
Non-trainable params: 0		

**Table 4:** Comparison of existing and proposed techniques of GoogLeNet model.

Approach	Dataset	Modality	Classification Type	Accuracy
Hosseini-Asl et al. [50]	210 subjects (70 AD, 70CAD-dementia NC, 70 MCI) ADNI	MRI/CT	Binary/Multi	AD vs. EMC vs. HC: 89.1% AD + MCI/NC: 90.3% AD/NC: 97.6% MCI/NC: 90.8%
Sahumbaiev et al. [51]	530 subjects (185 AD, 185, MCI, 160 HC) ADNI	MRI/CT	Multi	AD/MCI/NC: 88.31%
Korolev et al. [52]	50 AD, 43 LMCI, 77 EMCI, 61 NC- ADNI	MRI/CT	Binary	AD vs. NC: 80% AD vs. EMCI: 63% AD vs. LMCI: 59% LMCI vs. NC: 61% LMCI vs. EMCI: 52% EMCI vs. NC: 56%
Juan Ruiz et al. [53]	600 brain MRI images- ADNI	MRI/CT	Multi	AD, EMCI, LMCI, NC: 66.67%
Proposed *fine*-tuned GoogleNet model	300 subjects (75 AD, 75 EMCI, 75 LMCI, 75 NC)	MRI/CT	Multi	AD/EMCI/LMCI/NC: 98%

## Data Availability

The data presented in this study are available on request from the corresponding author.
